# Mechanisms and Evolution of Heritable Microbial Density in Insect Hosts

**DOI:** 10.1128/mSystems.00728-21

**Published:** 2021-08-31

**Authors:** Benjamin J. Parker

**Affiliations:** a Department of Microbiology, University of Tennessee, Knoxville, Tennessee, USA

**Keywords:** density, evolution, insect, mechanism, vertical transmission

## Abstract

Within-host density is a critically important aspect of vertically transmitted symbioses that influences the fitness of both hosts and microbes. I review recent studies of symbiont density in insects, including my laboratory’s work on pea aphids and maternally transmitted bacteria. These studies used systems approaches to uncover the molecular mechanisms of how both hosts and microbes influence symbiont density, and they shed light on whether optimal density is different from the perspective of host and microbial fitness. Mounting empirical evidence suggests that antagonistic coevolution shapes vertically transmitted symbioses even when microbes provide clear benefits to hosts. This is potentially because of differing selective pressures at the host and within-host levels. Considering these contrasting evolutionary pressures will be critically important in efforts to use vertically transmitted symbionts for biocontrol and as lessons from model systems are applied to the study of more complex microbiomes.

Invertebrates frequently associate with vertically transmitted symbionts, including bacteria, fungi, and viruses. Together, hosts and microbes perform essential biological functions that impact the fitness of both organisms. However, the line between mutualism and parasitism can be blurry for vertically transmitted symbioses. This is because microbes vary in their fitness effects on host organisms in a way that is often context dependent (e.g., protection against spatially or temporally variable pathogens). A critical aspect of the biology of these interactions is the within-host density of symbiont infections. For hosts, greater symbiont density can incur higher fitness costs (1, 2) but in some cases yields stronger benefits (3). For symbionts, density can influence transmission success (4) and also influences microbial fitness to the extent that it is linked to host fitness (5).

## WHICH PARTNER CONTROLS SYMBIONT DENSITY AND HOW?

Vertically transmitted symbionts have been found at variable densities within and among species (Box 1). Evolutionary genetic and systems approaches have been key to uncovering the mechanisms underlying this variation and how density evolves in natural populations. My lab is among several that study the association between pea aphids (Acyrthosiphon pisum) and bacteria that are transmitted from mothers to offspring. In addition to an obligate nutritional symbiont, aphids harbor several species of facultative symbionts that are not found in all individuals. Pea aphids form a species complex of reproductively isolated host plant-adapted populations, termed biotypes. Importantly, the frequency of facultative symbionts is variable across biotypes (reviewed in reference 6). Studies have found clear patterns of association between biotypes and specific symbiont species. For example, the Gram-negative facultative symbiont Regiella insecticola, which confers protection to aphids against specialist fungal pathogens (6), is strongly associated with the aphid biotype that lives on clovers. This association has been shown to be driven primarily by a specific phylogenetic clade of *Regiella*, referred to as clade 2 in most studies (7).

We found that genetic variation among both aphid biotypes and *Regiella* strains contributes to large differences in *Regiella* density. Higher density comes at a cost to aphid survival but provides no additional protection against fungi (1). In subsequent work, we have shown that aphids harboring *Regiella* decrease expression of innate immune system genes, including the key immune enzyme phenoloxidase, and we found that suppression of phenoloxidase via RNA interference (RNAi) leads to increased *Regiella* density (8). These data suggest that aphid innate immune mechanisms regulate symbiont density, complementing findings that aphid immune cells engulf *Regiella* (9). We have further found that “immune suppression” is especially strong in aphids harboring clade 2 *Regiella*, which reaches substantially higher densities in aphids and imposes stronger survival costs on hosts than other *Regiella* clades (1, 8).

Across systems, the relative importance of host versus microbial genotype in explaining variation in density varies considerably. For example, symbiont strain seems to be a more important factor than host genotype in the density of *Wolbachia* among *Drosophila* species (2). A *Wolbachia* strain from Drosophila melanogaster (*w*Mel) confers protection to its hosts against RNA viruses, and higher symbiont densities confer stronger antiviral protection (reviewed in reference 3). A variant of *w*Mel termed *w*MelPop was found to overproliferate within hosts, leading to reduced host life span, and within-host density is controlled by a region of the *w*Mel genome named Octomom through an unknown mechanism (10).

Variation in *Wolbachia* density can also be driven by host mechanisms. Symbiont densities vary across closely related species of *Nasonia* wasps, and a recent study used systems approaches to implicate a wasp gene in this interspecies variation. The function of the gene is currently unknown, but it is speculated that it could inhibit transmission between host cells or could act through the host immune system (11). Additional mechanisms of density regulation are being uncovered in systems beyond insects: for example, recent studies suggest that corals use nitrogen deprivation as a mechanism to control symbiotic algal densities (12).

## DOES OPTIMAL DENSITY DIFFER FOR HOSTS AND SYMBIONTS?

Hosts are under selection to accommodate and maintain associations with beneficial vertically transmitted symbionts, and they have evolved ways to house microbes and avoid clearing symbiont infections. Symbionts, in turn, have evolved adaptations to living within their hosts (e.g., 13). But within certain bounds, is the “optimal density” of a symbiont infection the same from the perspective of the host and microbe?

**FIG 1:**
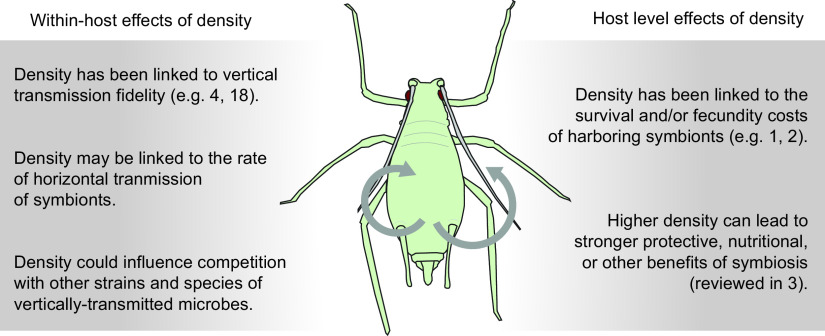
Host and within-host effects of density. The illustration summarizes potential within-host (left) and host-level (right) effects of symbiont density on the fitness of hosts and/or microbes. Numbers refer to published studies, as indicated.

Recent studies suggest not and that evolutionary conflict is an important factor in host-symbiont coevolution. As we have seen from several systems, the density of vertically transmitted symbionts is surprisingly variable. This is the case even in obligate nutritional symbioses that are required for host survival (14). Further, studies suggest that vertically transmitted symbiont density evolves under differing selective pressures at the host versus the within-host level (Fig. 1). For example, the Octomom region of *w*MelPop mentioned above has been shown to be highly unstable, with copy number increasing over the lives of individual flies (15). Despite the costs to host flies of high-density *w*MelPop infections, a recent experimental evolution study did not find any reduction of symbiont density or reduction of fitness costs to hosts. The experiment was conducted over 17 generations under laboratory conditions expected to select for reduced symbiont virulence (16). A plausible explanation for these findings is that within-host pressures select for symbionts with higher Octomom copy number despite the increased costs to hosts (15, 16).

In pea aphids, we have found that whether immune gene suppression occurs in hosts harboring a single strain of *Regiella* is variable across host plant-associated biotypes. As discussed above, the clover biotype has a close association with clade 2 *Regiella* (7). We used transcriptome sequencing (RNA-seq) to show that clover aphids do not experience any immune suppression when harboring clade 2 *Regiella* and harbor stable infections of these symbionts at lower densities than other biotypes (8). It is currently unclear if this strong association between clade 2 *Regiella* and clover aphids is a cause or consequence of these differences in immunity. Our data suggest that aphids do not benefit from higher *Regiella* densities (1)—fungal protection differs across *Regiella* clades but is dependent on specificity against certain fungal pathogen genotypes rather than density (1, 17). It therefore seems likely that preventing immune suppression and high symbiont density by clade 2 *Regiella* is adaptive in clover aphids.

Why might vertically transmitted symbionts benefit from increased within-host density at the expense of host fitness? Density has been linked to transmission fidelity in some systems—e.g., in naturally collected Drosophila innubila flies harboring variable densities of *Wolbachia* (4) and in laboratory D. melanogaster lines experimentally inoculated with different densities of *w*Mel (18). One possibility is that vertically transmitted microbes are subject to the same virulence/transmission tradeoffs as are important in pathogen evolutionary dynamics (5). Further, some mainly vertically transmitted symbionts are transmitted horizontally on evolutionary (7) and even ecologically (19) relevant timescales. A recent study found, for example, that parasitoid wasps can act as vectors of *Wolbachia* transmission: bacteria from infected whiteflies contaminate wasp ovipositors and transmit to new hosts upon nonlethal probing (20). It is possible that higher within-host density could increase the rate of this horizontal transfer, though more data are clearly needed. A last possibility is that increased density could help symbionts compete with other strains and species of microbes in host environments (5). Interestingly, a recent study using metagenome sequencing showed that aphids collected from the field can contain coinfections of multiple strains of *Regiella* (21). We are currently using competition assays to test the hypothesis that growing to higher within-host densities via immune suppression is a strategy adopted by clade 2 *Regiella* to outcompete other *Regiella* strains. Further, we are using genetic mapping and symbiont comparative genomics to uncover the mechanisms underlying variation in density. Interestingly, the immune genes that are suppressed during *Regiella* infections also play an important role in aphid resistance to fungal and bacterial pathogens, and a critical question to be addressed in our future work is how the immune system evolves in response to its dual roles of regulating beneficial microbial density while combatting pathogens.

## “HOLOBIONT” LEVEL FOCUS MAY MISS THE IMPORTANCE OF WITHIN-HOST DYNAMICS

Vertically transmitted symbionts have exciting potential for applied use, including with genetically engineered microbes (22). Further, the complex evolutionary dynamics studied in model systems are also likely to be relevant to more diverse host-associated microbial communities, in which vertical transmission is widespread (23). There has therefore been heightened recent interest in developing conceptual models of host-microbiome evolution, including integrated models that focus on hosts and their microbial symbionts as units of selection (i.e., holobionts). The findings discussed above, which emphasize the importance of within-host selection shaping symbiont genomes and the role of antagonistic coevolution between even highly integrated host-microbe pairings, do not lend support to the holobiont model. Systems approaches are expected to shed light on several critical questions about host-symbiont coevolution, including how the immune system evolves to accommodate and control complex microbiomes, how traditional mechanisms of microbial virulence evolve to play a role in symbiosis (13), and why some lineages of microbes but not others repeatedly form associations with host organisms. In this future work it will be critically important to consider both host- and within-host-level selection when studying microbiomes in organisms relevant to human health, agriculture, and conservation.

**BOX 1: HOW IS HERITABLE MICROBIAL DENSITY MEASURED?**

Many of the heritable microbes associated with insects are unculturable, and consequently, it can be difficult to measure symbiont within-host density. The most frequent approach involves the use of quantitative PCR (qPCR). Relative threshold cycle (*C_T_*) qPCR methods can be used to compare the amplification of symbiont and host genes in total genomic DNA samples (for an example, see reference 1), giving a relative measure of the ratio of symbiont to host cells among two or more treatments. “Standard curve” qPCR methods use a generated serial dilution of cloned PCR fragments (or, less ideally, quantified PCR product) to measure the number of copies of a symbiont gene in a specific amount of extracted DNA. The number of microbes can then be calculated using the total amount of DNA extracted from a host. Because DNA quantification (e.g., using a spectrophotometer) can be inaccurate, and because extraction methods do not recover all host genomic DNA, studies using standard curve methods often also measure copies of a host gene and report density as a ratio of symbiont to host cells.

Recently, systems approaches have been applied to the measurement of symbiont within-host density. For example, a recent study measured aphid obligate symbiont density across developmental stages using counts of symbiont reads in transcriptome data (24). Similarly, a recent study mined the NCBI Sequence Read Archive for *Wolbachia*, *Rickettsia*, and *Spiroplasma* reads and used these data to uncover substantial variation in symbiont density across taxa. This study further identified single nucleotide polymorphisms (SNPs) in Drosophila melanogaster hosts associated with variation in *Wolbachia* density (25).

## References

[1] Parker BJ, Hrcek J, McLean AHC, Brisson JA, Godfray HCJ. 2021. Intraspecific variation in symbiont density in an insect-microbe symbiosis. Mol Ecol 30:1559–1569. doi:10.1111/mec.15821. 33512733

[2] Martinez J, Tolosana I, Ok S, Smith S, Snoeck K, Day JP, Jiggins FM. 2017. Symbiont strain is the main determinant of variation in Wolbachia-mediated protection against viruses across Drosophila species. Mol Ecol 26:4072–4084. doi:10.1111/mec.14164. 28464440 PMC5966720

[3] Lopez-Madrigal S, Duarte EH. 2019. Titer regulation in arthropod-Wolbachia symbioses. FEMS Microbiol Lett 366:fnz232. doi:10.1093/femsle/fnz232. 31750894

[4] Unckless RL, Boelio LM, Herren JK, Jaenike J. 2009. Wolbachia as populations within individual insects: causes and consequences of density variation in natural populations. Proc Biol Sci 276:2805–2811. doi:10.1098/rspb.2009.0287. 19419989 PMC2839946

[5] Alizon S, de Roode JC, Michalakis Y. 2013. Multiple infections and the evolution of virulence. Ecol Lett 16:556–567. doi:10.1111/ele.12076. 23347009

[6] McLean AH, Parker BJ, Hrcek J, Henry LM, Godfray HC. 2016. Insect symbionts in food webs. Philos Trans R Soc Lond B Biol Sci 371:20150325. doi:10.1098/rstb.2015.0325. 27481779 PMC4971179

[7] Henry LM, Peccoud J, Simon JC, Hadfield JD, Maiden MJ, Ferrari J, Godfray HC. 2013. Horizontally transmitted symbionts and host colonization of ecological niches. Curr Biol 23:1713–1717. doi:10.1016/j.cub.2013.07.029. 23993843 PMC3980636

[8] Nichols HL, Goldstein EB, Saleh Ziabari O, Parker BJ. 2021. Intraspecific variation in immune gene expression and heritable symbiont density. PLoS Pathog 17:e1009552. doi:10.1371/journal.ppat.1009552. 33901257 PMC8102006

[9] Schmitz A, Anselme C, Ravallec M, Rebuf C, Simon JC, Gatti JL, Poirie M. 2012. The cellular immune response of the pea aphid to foreign intrusion and symbiotic challenge. PLoS One 7:e42114. doi:10.1371/journal.pone.0042114. 22848726 PMC3407134

[10] Chrostek E, Teixeira L. 2015. Mutualism breakdown by amplification of Wolbachia genes. PLoS Biol 13:e1002065. doi:10.1371/journal.pbio.1002065. 25668031 PMC4323108

[11] Funkhouser-Jones LJ, van Opstal EJ, Sharma A, Bordenstein SR. 2018. The maternal effect gene Wds controls Wolbachia titer in Nasonia. Curr Biol 28:1692–1702.e6. doi:10.1016/j.cub.2018.04.010. 29779872 PMC5988964

[12] Krueger T, Horwitz N, Bodin J, Giovani ME, Escrig S, Fine M, Meibom A. 2020. Intracellular competition for nitrogen controls dinoflagellate population density in corals. Proc Biol Sci 287:20200049. doi:10.1098/rspb.2020.0049. 32126963 PMC7126079

[13] Enomoto S, Chari A, Clayton AL, Dale C. 2017. Quorum sensing attenuates virulence in Sodalis praecaptivus. Cell Host Microbe 21:629–636.e5. doi:10.1016/j.chom.2017.04.003. 28494244 PMC5542680

[14] Chong RA, Moran NA. 2016. Intraspecific genetic variation in hosts affects regulation of obligate heritable symbionts. Proc Natl Acad Sci USA 113:13114–13119. doi:10.1073/pnas.1610749113. 27799532 PMC5135297

[15] Chrostek E, Teixeira L. 2018. Within host selection for faster replicating bacterial symbionts. PLoS One 13:e0191530. doi:10.1371/journal.pone.0191530. 29346449 PMC5773213

[16] Monnin D, Kremer N, Michaud C, Villa M, Henri H, Desouhant E, Vavre F. 2020. Experimental evolution of virulence and associated traits in a Drosophila melanogaster–Wolbachia symbiosis. bioRxiv. doi:10.1101/2020.04.26.062265.

[17] Parker BJ, Hrcek J, McLean AHC, Godfray HCJ. 2017. Genotype specificity among hosts, pathogens, and beneficial microbes influences the strength of symbiont-mediated protection. Evolution 71:1222–1231. doi:10.1111/evo.13216. 28252804 PMC5516205

[18] Liu XC, Li ZX. 2021. Transmission of the wMel Wolbachia strain is modulated by its titre and by immune genes in Drosophila melanogaster (Wolbachia density and transmission). J Invertebr Pathol 181:107591. doi:10.1016/j.jip.2021.107591. 33882275

[19] Duron O, Wilkes TE, Hurst GD. 2010. Interspecific transmission of a male-killing bacterium on an ecological timescale. Ecol Lett 13:1139–1148. doi:10.1111/j.1461-0248.2010.01502.x. 20545734

[20] Ahmed MZ, Li SJ, Xue X, Yin XJ, Ren SX, Jiggins FM, Greeff JM, Qiu BL. 2015. The intracellular bacterium Wolbachia uses parasitoid wasps as phoretic vectors for efficient horizontal transmission. PLoS Pathog 10:e1004672. doi:10.1371/journal.ppat.1004672. 25675099 PMC4347858

[21] Guyomar C, Legeai F, Jousselin E, Mougel C, Lemaitre C, Simon JC. 2018. Multi-scale characterization of symbiont diversity in the pea aphid complex through metagenomic approaches. Microbiome 6:181. doi:10.1186/s40168-018-0562-9. 30305166 PMC6180509

[22] Elston KM, Leonard SP, Geng P, Bialik SB, Robinson E, Barrick JE. 5 June 2021. Engineering insects from the endosymbiont out. Trends Microbiol doi:10.1016/j.tim.2021.05.004. 34103228

[23] Funkhouser LJ, Bordenstein SR. 2013. Mom knows best: the universality of maternal microbial transmission. PLoS Biol 11:e1001631. doi:10.1371/journal.pbio.1001631. 23976878 PMC3747981

[24] Pers D, Hansen AK. 2021. The boom and bust of the aphid’s essential amino acid metabolism across nymphal development. G3 (Bethesda) doi:10.1093/g3journal/jkab115. PMC8433001 33831149

[25] Medina P, Russell SL, Aswadhati K, Corbett-Detig R. 2020. Deep data mining reveals variable abundance and distribution of microbial reproductive manipulators within and among diverse host species. bioRxiv doi:10.1101/679837. PMC10335654 37432953

